# Esad Feyzi (1874-1902): Turkey's Pioneering Radiologist

**DOI:** 10.7759/cureus.75784

**Published:** 2024-12-16

**Authors:** Yasemin Koçer Tulgar, Meltem Dogan

**Affiliations:** 1 Medical History and Ethics, Samsun University, Samsun, TUR; 2 First and Emergency Aid, Istanbul Şişli Meslek Yüksekokulu, Istanbul, TUR

**Keywords:** esad feyzi, historical vignette, medical history, radiology, x-rays

## Abstract

Dr. Esad Feyzi was a pioneering physician who lived in the 19th-century Ottoman Empire and made significant contributions to the field of medicine. Despite passing away at the young age of 28, he achieved notable scientific advancements during his lifetime. Most prominently, he developed the first X-ray device in his country despite the limited resources available to him. Alongside his classmate, Dr. Rıfat Osman, he was among the first to utilize radiological applications in the treatment of wounded soldiers during wartime, a feat accomplished while he was still a student. In this context, Dr. Esad Feyzi is regarded as a significant leader in the field of radiology within Turkish medical history, influencing advancements in this field up to the present day. The purpose of this article is to provide a brief introduction to the life of Dr. Esad Feyzi.

## Introduction and background

The discovery of X-rays revolutionized both medical diagnostics and therapeutic techniques, marking a turning point in medicine and physics. Wilhelm Conrad Röntgen's groundbreaking identification of X-rays in 1895 heralded a new era in scientific and medical advancements. As a German physicist, Röntgen initially observed this novel form of radiation without fully comprehending its nature. However, he swiftly recognized its capacity to penetrate matter, enabling him to capture the first radiographic image of a human hand's bone structure. This discovery not only transformed the physical sciences but also initiated profound changes in medical practices, ultimately establishing the foundation of radiology as a distinct discipline. In 1901, Röntgen was awarded the inaugural Nobel Prize in Physics in recognition of his monumental contributions. This award underscored the enduring significance of his work within the scientific and medical communities. His discovery laid the groundwork for contemporary diagnostic and therapeutic innovations, cementing his legacy as a pivotal figure in medical history [1]. 

The global impact of X-rays extended to the Ottoman Empire, where Esad Feyzi emerged as a pioneering figure in integrating this transformative technology into medical practice. Dr. Esad Feyzi's contributions significantly advanced the field of radiology within the region. This study aims to explore Dr. Esad Feyzi's life and his seminal contributions to the development of medical radiology. 

## Review

Life and career

Esad Feyzi is widely acknowledged as a pioneering figure among Ottoman medical professionals for his innovative application of X-ray technology in battlefield medicine. Born in 1874 in the district of Bursa/Gemlik, located in present-day Turkey, Esad Feyzi demonstrated an early aptitude and profound interest in the natural sciences. His exceptional abilities in this field set him apart, laying the foundation for his significant contributions to medical science.

Esad Feyzi's passion for scientific inquiry drove him to pursue advanced knowledge in medicine, where he became a prominent advocate for integrating modern methodologies into Ottoman medical practice. His progressive approach combined contemporary technologies with traditional medical techniques, establishing him as a visionary in his field. Notably, his pioneering use of X-rays for medical diagnostics and treatment on the battlefield represents a transformative moment in the history of Turkish medicine, underscoring his courage and scientific acumen [2].

**Figure 1:**
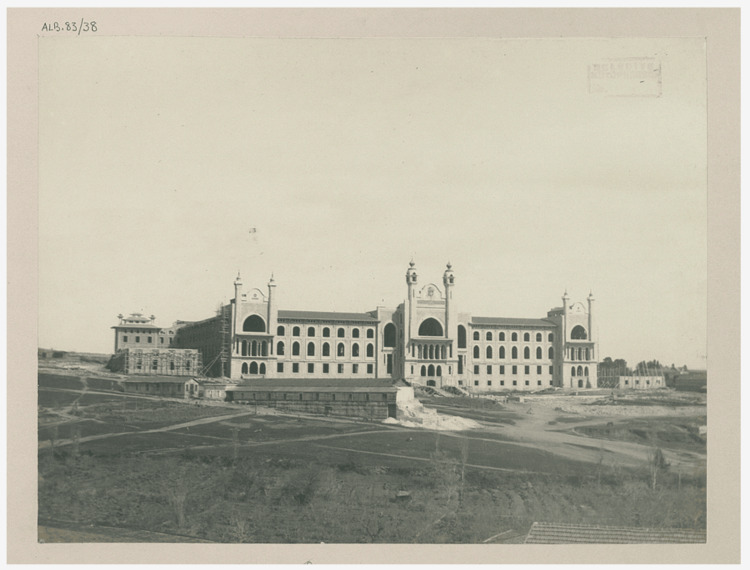
Mekteb-i Tıbbiye-i Şahane. Permission obtained from Istanbul Atatürk Library, (Photograph by Ali Sami) [4].

Esad Feyzi's education process started in Istanbul, and he continued by receiving secondary school education at Davutpaşa Military Junior High School. After this basic education period, he entered the Medical Preparatory School and was later accepted to the Military Medical School (Figure 1). During this period, Esad Feyzi successfully merged his deep interest in the natural sciences with his medical education, making significant contributions to both scientific and medical fields. His pioneering efforts in the medical application of X-rays under wartime conditions, making him one of the first in the Ottoman Empire to implement this technology, secured his place in history. This period highlights his ability to effectively integrate scientific knowledge with medical practice, showcasing his innovative approach within the medical field [3].

The initial applications of X-rays in the Ottoman Empire began in 1896 under the efforts of Monseigneur Isoard (also referred to as Isoire), a physics and mathematics teacher at Galatasaray High School. Isoard conducted a series of experiments to explore the potential of X-rays, one of the most groundbreaking scientific innovations of the era. His first successful experiment involved capturing the image of a metal coin inside a pouch using X-rays. Subsequently, Isoard expanded his studies to human anatomy, producing an X-ray image of his son's hand. These experiments highlighted the dual potential of X-rays for imaging both inanimate objects and human anatomy, paving the way for their application in medical science [5].

During the same period, Mr. Halit, a photographer based in Istanbul, demonstrated an interest in the emerging X-ray technology. Through his experiments, he successfully produced an X-ray image of a pen, showcasing the adaptability of this technology for visual arts and photography. These initial explorations by Isoard and Halit laid the groundwork for the scientific and medical adoption of X-rays in the Ottoman Empire, marking a significant step toward further advancements in the field [3].

In the latter part of 1896, Dr. Esad Feyzi Bey initiated the first medical applications of X-rays at the Mekteb-i Tıbbiye-i Askeriye-i Şahane (Imperial Military Medical School) in Istanbul, Demirkapı. Utilizing a radiographic device he designed with the limited technological resources available, Dr. Esad Feyzi conducted experiments that represented the earliest steps in the integration of radiology into modern medicine in Turkey. His independent initiatives in radiography were instrumental in advancing medical education and practice. Subsequently, he earned recognition as an Associate Professor of Physics and Radiological Specialist in Surgical Diseases at the *Mekteb-i Tıbbiye-i Şahane*. His pioneering contributions are regarded as a cornerstone in the evolution of Turkish diagnostic methods, solidifying his legacy as a leading figure in Turkish medical history (Fıgure 2). In 1897, following the outbreak of the Turkish-Greek War, Dr. Esad Feyzi and his colleague, Dr. Rıfat Osman Tosyalı (1874-1933), transitioned their X-ray work to a professional setting, becoming some of the earliest practitioners to apply radiology in wartime conditions. This marked a critical moment in the advancement of radiology as a tool for military medicine [5-7,8].

**Figure 2:**
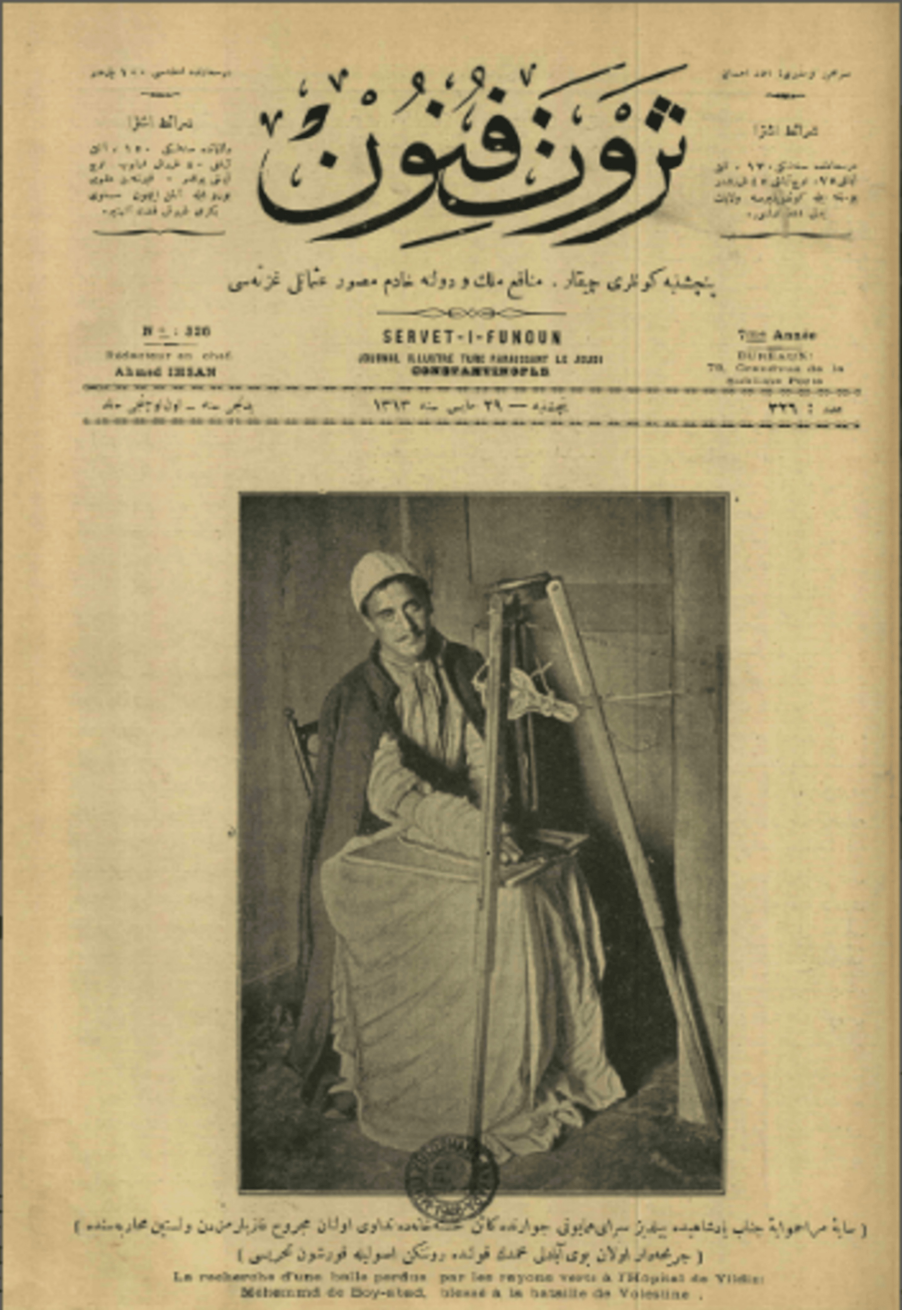
Soldier Boyabatlı Mehmet. Permission received from Boğaziçi University, Turkish language and literature department Servet-i Fünun digital journal (Tubitak Project) [8].

During the later stages of the war, injured soldiers were evacuated from the Greek front to the Yıldız Hamidiye Sultan Military Hospital in Istanbul for treatment. Dr. Esad Feyzi and Dr. Rıfat Osman approached the hospital's chief physician and their mentor, Dr. Cemil Topuzlu (1866-1958), proposing the use of X-ray technology to locate shrapnel fragments in wounded soldiers and assist in surgical interventions. After evaluating their proposal, Dr. Cemil Topuzlu obtained special permission from Sultan Abdülhamid Khan II to enable the use of X-ray devices for this purpose. One of the notable outcomes of their work was the radiographic imaging of the hand of a wounded soldier named Boyabatlı Mehmed. These images were subsequently published in the renowned art and literature journal Servet-i Fünun, marking the first documented radiological images of wartime injuries in Turkey. This groundbreaking achievement is recognized as a significant milestone in the integration of radiology into wartime medical practices and one of the earliest examples of its application in medical history [2,9].

Thus, the radiological work carried out at the Yıldız Mobile Military Hospital emerged as a significant innovation in the medical practice of the time [2,3,9].

Dr. Esad Feyzi’s works

Despite his short life, Dr. Esad Feyzi made profound contributions to science and medicine, leaving an enduring legacy through his academic works. His scientific impact is evidenced by two major books and several influential articles. His first significant publication, İlmü’l-Arz ve’l-Ma’âdin, focused on geology and mineralogy and served as a foundational resource for students in these disciplines. This work was deemed so valuable that it was published twice. Another noteworthy contribution was his book Röntgen Şu’â’tı ve Tatbikât-ı Tıbbiye ve Cerrahiyesi, which provided a comprehensive analysis of X-rays and their medical applications. This publication played a crucial role in the early understanding and dissemination of radiological techniques in medicine.

Additionally, Dr. Esad Feyzi authored a pioneering article on X-rays titled Röntgen şu’a’â-tının sûret-i istihsâli, havvâsı, mâhiyeti, tatbikât-ı tıbbiyesi, which was published in the journal Nevsâl-i Âfiyet. This article offered detailed insights into the production, properties, and medical applications of X-rays, contributing significantly to the scientific knowledge of the time [3].

Dr. Esad Feyzi’s academic achievements earned him widespread recognition throughout his career. He graduated in 1897 from the esteemed Mekteb-i Tıbbiye-i Şahane (Imperial Medical School) as one of the institution's most accomplished students. Although he was initially assigned to Yemen in the post-graduation placement lottery, he chose to remain in Istanbul following the advice of his mentors-a decision that proved pivotal for his career. In Istanbul, he began working as an assistant in physics at the Military Medical School, where he embarked on groundbreaking scientific work. Shortly after, he joined the faculty of the Mekteb-i Mülkiye-i Şahane (Civil Medical School), where he taught geology and mineralogy, imparting his expertise to a new generation of students. These academic endeavors underscore Dr. Esad Feyzi’s visionary approach to science and his significant contributions to medical education during this period [2,3].

Tragically, Dr. Esad Feyzi’s promising career was cut short. Just four years after his graduation, he contracted erysipelas on his face, which progressed to meningitis. This illness led to his untimely death in 1902 at the age of 28, robbing the medical field of one of its most promising scientists. Despite his brief life, Dr. Esad Feyzi's pioneering work continues to be remembered as a cornerstone in the early development of medical radiology and scientific education in Turkey [2,3,9].

## Conclusions

During his short life, Dr. Esad Feyzi made significant contributions to radiological medicine and established himself as a pioneer in Turkish medical history. His innovative work in radiology greatly contributed to the progress of 19th-century Turkish medicine, paving the way for advancements in medical science and the widespread adoption of radiological techniques. Feyzi’s scientific endeavors played a critical role in the modernization of Turkish medicine, providing a strong foundation for future medical practices.
